# The neurocognitive mechanism linking temperature and humidity with miners’ alertness: an fNIRS study

**DOI:** 10.1038/s41598-024-62674-z

**Published:** 2024-05-23

**Authors:** Chenning Tian, Hongxia Li, Shuicheng Tian, Fangyuan Tian, Hailan Yang

**Affiliations:** 1 Institute of Safety Management and Risk Control, School of Safety Science and Engineering, Xi’an University of Science and Technology, Xi’an, 710054 China; 2 Institute of Safety and Emergency Management, School of Safety Science and Engineering, Xi’an University of Science and Technology, Xi’an, 710054 China; 3 School of Management, Xi’an University of Science and Technology, Xi’an, 710054 China

**Keywords:** Environmental impact, Attention, Health occupations

## Abstract

As the depth of coal mining increases, the temperature and humidity of the underground environment also rise, which can negatively impact the physiological health of miners, and may even pose a threat to their safety and lives. However, studies on the neurocognitive mechanisms underlying the relationship between temperature, humidity, and miners’ alertness are scant. This study investigates several research objectives: (A) the differences in reaction time and error rate in different temperature and humidity conditions, which factor has a greater impact; (B) the differences in the levels of Oxy-Hb in different conditions and which factor has a greater impact; (C) the differences of activation degree between different regions of interest; and (D) the differences in the shape of Oxy-Hb time course between different conditions between different regions of interests. The fNIRS was used to measure the activity in 100 participants’ prefrontal cortex in this study. The results showed that both temperature and humidity would lead to decreased alertness of miners, which would not only prolong the reaction time, increase the error rate, and increase the Oxy-Hb concentration, but also lead to increased activation of the prefrontal cortex and greater activation of the right side than that of the left side, the Oxy-Hb time course was different on both sides, and temperature has a greater effect on alertness than humidity.

## Introduction

Since the onset of twenty-first century, considering the escalating need for coal and the exhaustion of superficial coal reserves, deep mining will become an important resource development strategy and the main way to ensure the sustainable development and supply of China’s mineral resources in the future^1,2^. With the increasing depth of coal mining, the underground ambient temperature also increases^3^. The hazards posed by elevated temperatures and humidity represent one of the most significant challenges to coal mining safety, which restricts coal mining activities^4,5^. The average mining depth in China has exceeded 650 m, where the average temperature of the original rock lies between 35.9 and 36.8 °C. There are more than 100 mines with depths greater than 700 m^6^, and upward of 80 mines exceed a depth of 1000 m^7^. Deep mining is also common in other countries. For example, the mining depth of the mine in Ibbenbüren, Germany, reached 1530 m before it stopped production, and the temperature of the underground rock can reach 60 °C. The mining depth of the President Steyn Gold Mine in South Africa has exceeded 3000 m, and the rock temperature is higher than 63 °C. High temperature in deep mines will not only affect the mechanical properties of surrounding rock but also have implications for the safe operation of mining activities^8^. Previous research has indicated that the physiological well-being of miners is severely compromised and the prevalence of specific diseases increases markedly heightened when the mine temperature surpasses 28 °C. Additionally, a 1 °C elevation in temperature results in a 6–8% reduction in the productivity of miners. When the working environment temperature exceeds 35 °C, the labor efficiency of miners is only 20% of that under normal conditions^9^. When miners need to react to new situations, fatigue caused by heat exposure can interfere with their ability to react quickly. Because the mining industry relies on heavy mobile equipment and other dangerous activities, such as detonating explosives, miners’ heat, sleepiness, and cognitive impairment are potential dangers to themselves and others^10^.

In the study of coal mine safety, human safety behavior has consistently been a focal point of research, and alertness is intimately connected with safety behavior. Reduced cognitive abilities such as alertness and reaction time can lead to fatigue, reduced work performance, unsafe behavior, and accidents^11,12^. To strengthen the management of miners’ safety behavior, it is necessary to study the influence of external environmental factors on miners’ neurocognitive function, so as to deeply understand the internal mechanism of miners’ unsafe behavior. In recent years, more and more scholars have begun to pay attention to the influence of temperature and humidity on cognitive function. As early as 1964, Bell et al. studied the performance of visual and auditory alertness tasks exposed to 29.5/24.5 °C (85/76°F) db/wb to 63/47 °C (145/117°F) db/wb under two climatic conditions. The results showed that in both experiments, more signals were missed as body (oral) temperature rose^13^. In 1992, Norin conducted a study on the alertness of drivers to the temperature in the compartment, and the results showed that in a relatively high temperature environment, the driver will miss more than 50% of the signal and the reaction time will be reduced by 22%^14^. Song et al. computed the spatial correlation amongst BOLD frequency and cerebral blood flow in various cerebral areas for every participant under two distinct thermal circumstances (50 °C and 25 °C). Increased BOLD frequency in the thalamus and ventromedial prefrontal cortex is correlated with increased reaction time at high temperatures compared to normal body temperature^15^. Other researchers have studied the relationship between skin temperature and alertness^16–19^. In general, there is still a lack of research investigating the alertness of miners under different temperature and humidity conditions from a neurocognitive perspective, utilizing fNIRS.

Psychologists have traditionally used self-report methods and performance on laboratory tasks to understand and predict human behavior. However, these indicators are only a limited predictor of behavior in specific situations. In contrast, neuroimaging can be used as a supplement to reveal links between neural activity and medium- and long-term, ecologically valid outcomes in laboratory settings^20,21^. Functional near-infrared spectroscopy (fNIRS) is an promising non-invasive imaging methodology which has garnered increasing popularity as a neuroimaging technique for cerebral function research in recent years^22–24^. Compared to functional magnetic resonance imaging (fMRI), this technology has several advantages, such as portability, extensive long-term data acquisition, and a high sample rate. For this reason, fNIRS has been widely used to locate brain activation during tasks^25,26^. In the field of safety research, scholars from various domains such as driving^27^, construction^28^, and aviation^29^ have extensively employed fNIRS to investigate workers’ unsafe behaviors.

Therefore, in order to gain a comprehensive understanding of the neurocognitive mechanisms behind miners’ unsafe behaviors, the study should focus on linking the temperature and humidity as well as the miners’ alertness. In this study, fNIRS was used to assess the performance of alertness in one hundred miners’ when exposed to varied temperatures and humidity condition. This study introduces a novel approach to investigating the relationship between the research environment and cognition, which aids in furthering the inter-disciplinary integration of neuroscience and coal mine safety sciences.

## Materials and methods

### Participants

A total of 100 miners were recruited to participate in the experiment, they were distributed among the four groups at 25 participants. The participants’ ages ranged from 26 to 42 years. All participants were male (In China, most miners are male, and women are prohibited working in underground mines), right-handed, and had no bad habits. Normal vision or visual acuity, no color blindness or other eye diseases. All the participants made sure they got adequate sleep and did not take psychotropic medication before the experiment. Each participant took part in the experiment in the morning to reduce the impact of circadian rhythms on alertness. Before the experiment was started, participants were told about its goal but not about the experimental design or the groupings. The right to withdraw was given to each participant prior to the experiment; however, no withdrawals took place. This study received ethical approval from the Ethics Committee of Xi’an University of Science and Technology and adhered to the latest version of the “Declaration of Helsinki.” Prior to performing any procedures in this study, written informed consent was obtained from each participant.

After removing the bad data caused by equipment failure or heavy head movement, the fNIRS data of 89 miners were obtained in this experiment. Participants with more than 20% faulty channels were excluded from consideration.

### Experimental environment and condition

**Figure 1:**
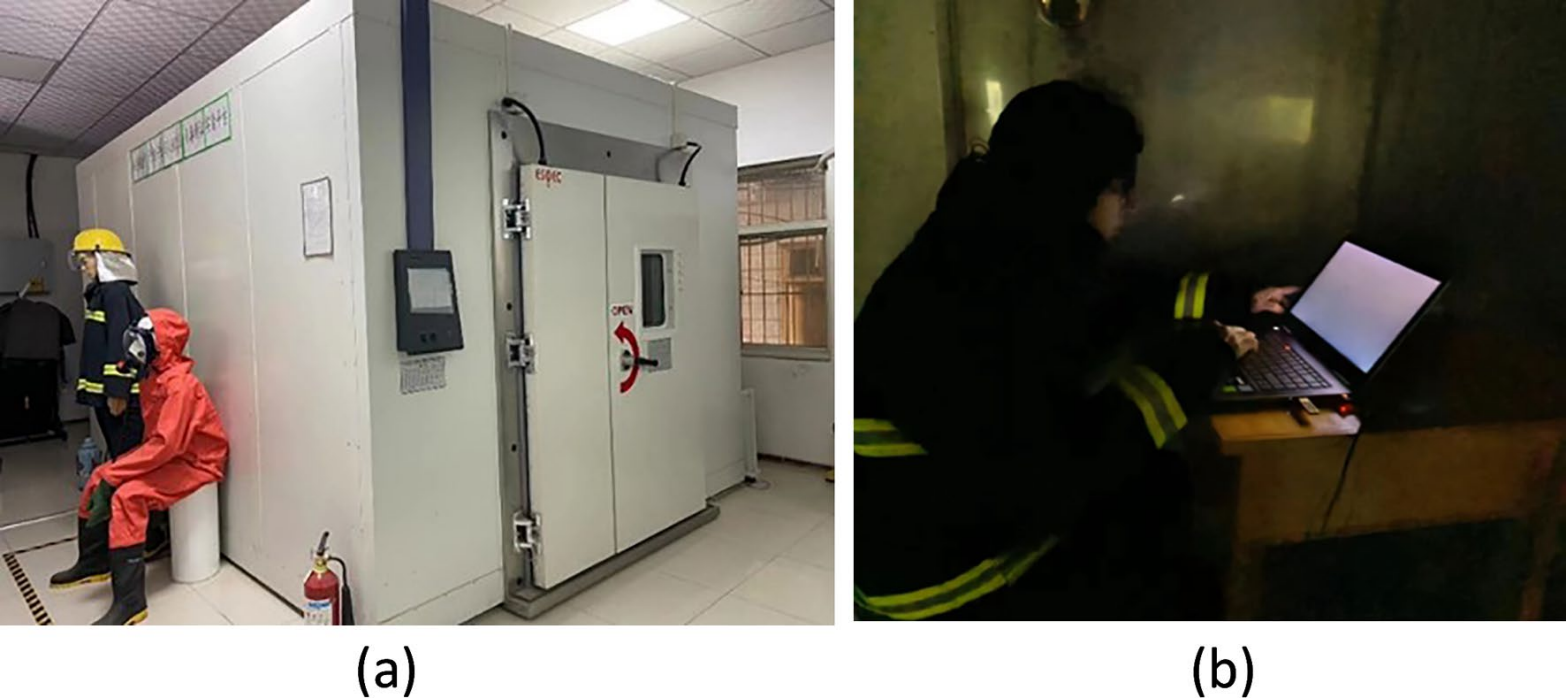
The experimental cabin and experimental scene. (**a**) The temperature and humidity cabin, (**b**)The experimental scene.

The experimental location was chosen in Safety and Emergency Management Laboratory of Xi’an University of Science and Technology. A walk-in temperature and humidity cabin (SEWTH-A-290H) was built in the laboratory. The interior dimension of the cabin is 3 * 2.4 * 4 (m), and the external dimension is 3.2 * 2.7 * 5.6 (m). Temperature setting ranges from − 20 to 90 °C, humidity control ranges from 20 to 95% R.H. During the experiment, the lights inside the cabin are off, and the only light source is the screen light of the experimental equipment. The screen light source was measured by a handheld illuminator (SuWei SW6023) with a brightness of 60 lx at the screen. The subjects sat in front of the only table (1 × 0.5 × 0.7 m) in the experimental cabin and performed the experimental tasks through the experimental machine on the table (screen size 19 inches; screen ratio 16:10; resolution 1680 × 1050). The average cabin noise volume is 71 decibels. Figure 1 shows the experimental environment and the experimental scene.

In the revised 2016 edition of the “Coal Mine Safety Regulations of China”, it is stated that when the air temperature on the mining face exceeds 26 °C and the temperature in the equipment chamber exceeds 30 °C, the working hours of personnel at overheated locations must be shortened, and thermal stress management measures must be taken. Compared to the maximum allowable temperatures in coal mines in other countries around the world, the environmental evaluation criteria in China’s coal mines are stricter. Statistics on underground working place humidity in China^30^ showed that the average relative humidity of coal mine working faces is 89%.

According to the actual temperature and humidity conditions in Chinese coal mines and the maximum working environment parameters supported by experimental measurement equipment, the specific experimental environment settings are presented in Table 1.

**Table 1:** Experimental conditions and participants information.

Conditions	Air temperature	Relative humidity (%)	Number of participants	Mean age
Condition I	25 °C (77℉)	55	24/25	32.52(± 4.38)
Condition II	35 °C (95℉)	55	20/25	32.32(± 4.86)
Condition III	25 °C (77℉)	85	20/25	33.16(± 5.33)
Condition IV	35 °C (95℉)	85	25/25	32.68(± 4.48)

“Number of Participants” refers to the number of valid participants/actual participants.

### Psychomotor vigilance task (PVT)

The psychomotor vigilance task (PVT) is a simple testing task used to evaluate and measure the time it takes for participants to respond to irregularly presented stimuli. In this task, participants are tasked with responding to each stimulus occurrence and tracking their reaction time (RT). Even when participants perform the same task at different times, the reaction time in the PVT can vary due to their psychological and physiological factors. The versatility of the PVT makes it a valuable instrument for studying various domains, including circadian rhythms and sleep research.

Currently, the utilization of fNIRS for studying brain correlates of PVT is relatively limited. Hall et al. employed the PVT to investigate attention deficits in preterm adolescents^31^. Hiroyasu et al. demonstrated the presence of hemodynamic differences between sensory modalities in the PVT task. They also found differences between the PVT task and the GO/NOGO sustained attention task^32^. Borragán et al. utilized the PVT to investigate frontal connectivity and sleep deprivation in young, healthy individuals^33^. However, to date, no studies have directly compared the hemodynamic differences induced by PVT under varying temperature and humidity conditions.

The aim of this study is to replicate the experimental paradigm established by Drummond^34^ using fNIRS. Building on these results, we investigated how well miners perform in terms of alertness under various humidity and temperature conditions. Our objectives were to assess: (A) the differences in reaction time and error rate in different temperature and humidity conditions, which factor has a greater impact; (B) the differences in the levels of Oxy-Hb in different temperature and humidity conditions and which factor has a greater impact; (C) the differences of activation degree between different regions of interest; and (D) the differences in the shape of Oxy-Hb time course between different conditions between different regions of interests. We hypothesize that: (A) different temperature and humidity conditions will result in differences in reaction time and error rate, with temperature having a greater impact than humidity; (B) different temperature and humidity conditions will lead to differences in Oxy-Hb changes, there are differences in the left and right prefrontal cortex. Additionally, we anticipate that it will be possible to observe different shapes of Oxy-Hb time course in different ROIs under different conditions, similar to the different brain/cognitive functions proposed by Dosebanch et al.^35^.

### Experimental procedures

**Figure 2:**
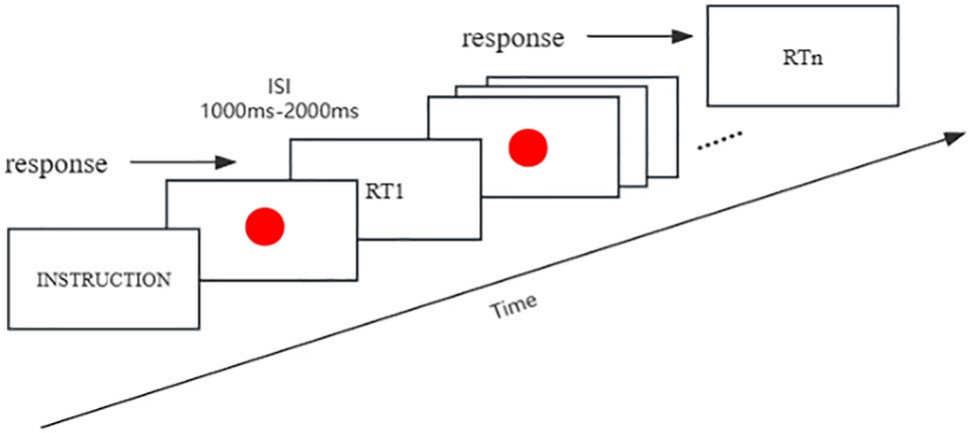
An illustration of the PVT experimental task.

**Figure 3:**
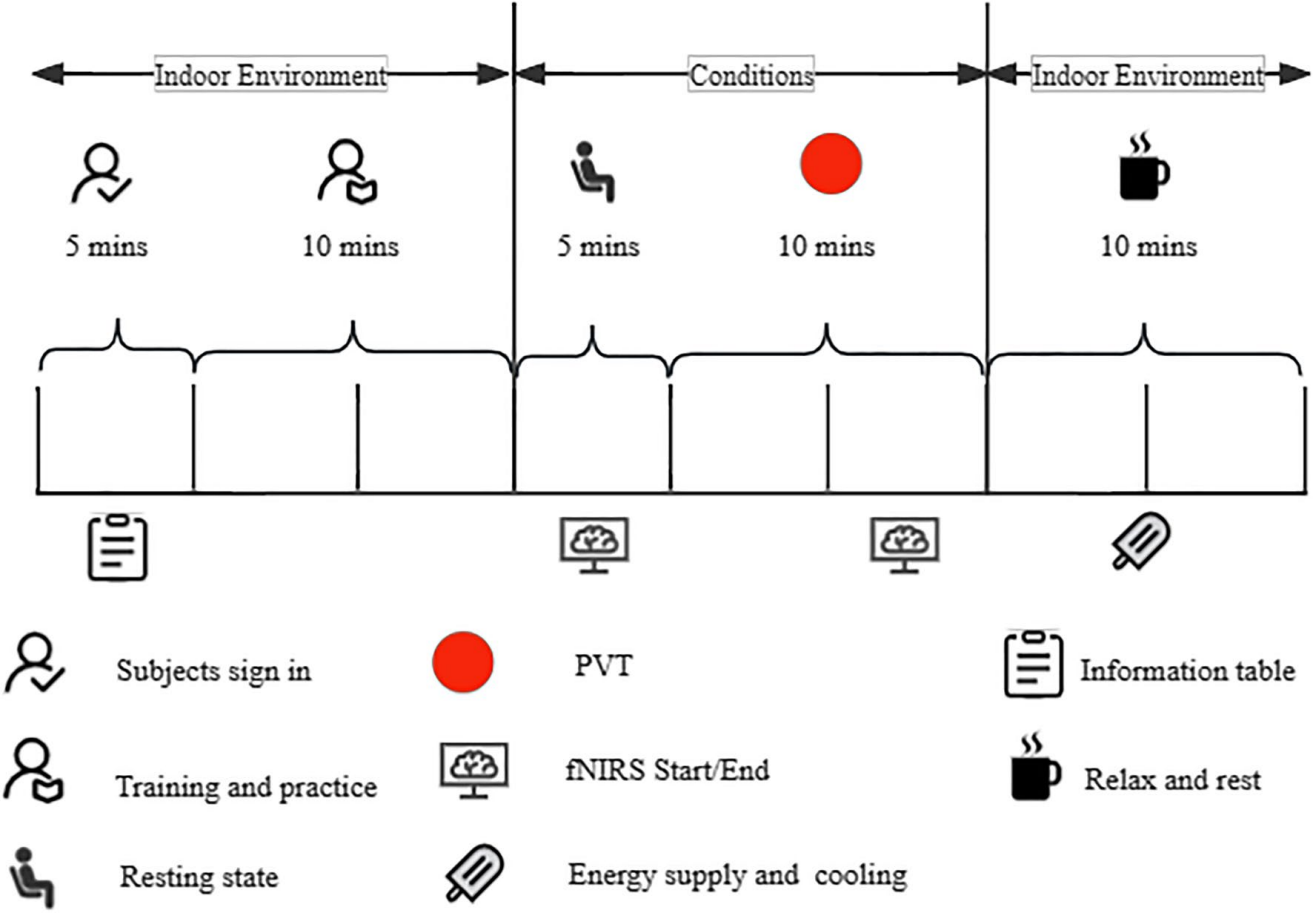
An illustration of the experimental process.

The PVT utilized in this study was programmed using E-Prime 3.0 software. After task initiation, a red dot randomly appeared in the center of a black background, with intervals ranging from 1000 to 2000 ms. Participants were required to press the space bar as soon as they observed the red dot. Once the red dot disappeared, the background program automatically recorded the duration from its appearance to disappearance, representing the participant’s RT. As the standard hemodynamic response takes approximately 10 s to return to the baseline level after reaching its peak, a minimum interval of 10 s or longer was implemented between each trial. Therefore, the red dot appeared with a random inter-stimulus interval ranging from 10 to 20 s. The illustration of the experimental task, experimental process are depicted in Figs. 2 and 3.

### Data acquisition

**Figure 4:**
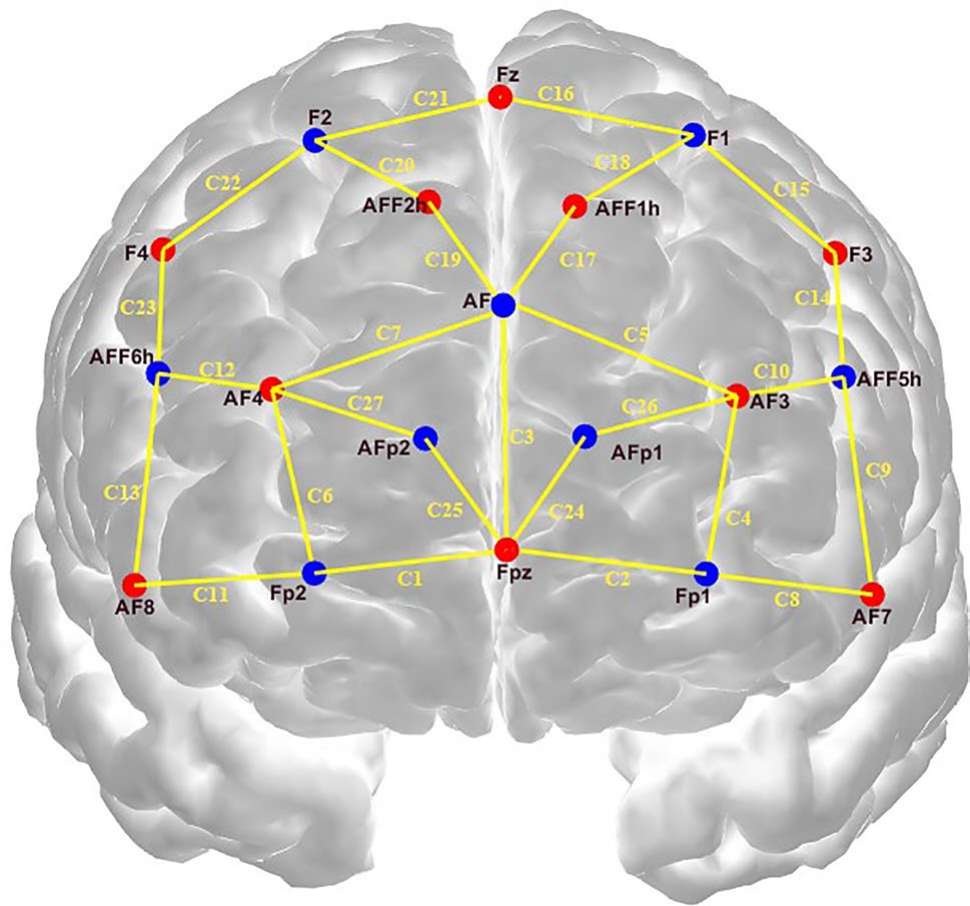
Distribution of optical electrodes. The source position is red and the detector is blue. The yellow line indicates the location of the fNIRS channel.

This experiment utilized the Cortivision Photon Cap, a portable near-infrared optical brain imaging system. This device, in conjunction with the Cortivision Pathfinder, measured hemodynamic data in the cerebral cortex. The Cortivision Photon Cap used a more detailed version of the “10–5 system”^36^, which goes beyond the “10–20 system” and “10–10 system” electrode placement rules that are usually used in electroencephalography (EEG) to pinpoint exactly where a problem is. This experiment covered a total of 27 channels near the prefrontal cortex. These channels consisted of 10 light sources and 9 detectors. The spacing between the light sources and detectors for all data channels was maintained at approximately 30 mm, while the short-distance channels had a fixed spacing of 20 mm. The sampling rate was 5–6 Hz. Figure 4 illustrated the distribution of optical electrodes. Table 2 provides information on the Brodmann region, anatomical location, and defined regions of interest (ROIs) used in this study, which excluded short-distance channels. BA 9, 10, 11 together form the prefrontal cortex, which is responsible for performing cognitive functions, including all aspects of thinking and intuition, memory and recall of information, problem solving, emotions, etc., and is closely linked to the limbic part of the forebrain.

**Table 2:** Channel distribution, Brodmann areas and anatomic location of ROIs.

Channels	Brodmann areas	Anatomic location	ROIs
1,3,6,7,11,13,20,21,22,27	9,10,11	Prefrontal cortex (right)	A
2,3,4,5,8,9,15,16,18,26	9,10,11	Prefrontal cortex (left)	B

### Informed consent

Informed consent was obtained from all subjects involved in the study. Written informed consent has been obtained from the patient(s) to publish this paper.

## Data analysis

### Behavioral data

For each participant, we collected their reaction times (RT) and the number of errors. Errors were defined where the reaction time exceeded 500 ms. We calculated the error rate (ER) as the ratio of the number of errors to the total number of trials. Tukey’s HSD test was performed on RT and ER of four groups to verify the effects of temperatures and humidity. To further substantiate that temperature exerts a greater impact on miners’ RT and ER than humidity does, employ a multiple linear regression model to analyze the RT and ER of the four conditions. The multiple linear regression model was created by MATLAB, temperature and humidity were extracted as independent variables, RT and ER were separately extracted as dependent variables. Additionally, an Analysis of Covariance (ANCOVA) analysis was performed to examine how age influences RT and ER independently of temperature and humidity.

### fNIRS data

Data were preprocessed using the NIRS_KIT software (version 3.0_Beta)^37^. During the data preparation step, data type is designated as type13 and the PPF value is set to [6 6], the modified Beer–Lambert law was applied to convert the raw light intensity data of the remaining channels into changes in oxygenated hemoglobin and deoxygenated hemoglobin concentrations^38^. Detrending was done in the first step towards the data, a polynomial regression model is used to estimate a linear or non-linear trend and then subtracts it from the raw hemoglobin concentration signal. In the second step, motion correction of fNIRS data was performed using the time derivative distributed repair (TDDR) method^39^. The third step uses a filter based on the fast Fourier transform (0.01–0.1 Hz) to transform the time series of each channel into the frequency domain, filter the frequency domain signal, and then transform back into the time domain. Finally, shallow noise is recorded using a short-range reference channel (sensitive only to signals from shallow layers of tissue outside the brain), and then noise is removed from the neural record using regression.

After the fNIRS data were preprocessed, individual-level statistical analyses were performed based on a general linear model (GLM) to examine task-related neural activation. GLM enables the calculation of the BOLD hemodynamic response function (HRF) from a linear combination of different components, providing a more accurate characterization of brain activation responses^40^. The following steps are included (1) the type of signal to be analyzed is selected as Oxy, changes in Oxy-Hb concentration will be used as a primary indicator of brain activity because they are less prone to confusion than Dxy-Hb^41^; hemodynamic response function (HRF): the default canonical HRF;(2) The matrix was designed according to the stimulus presentation time and reaction time of each subject, and “different” was selected in design Inf type, and then the designed matrix information was imported. In the design matrix, each row represents each time point, and each column represents an effect or an explanatory variable; (3) use contrast vector to estimate the mean value of the signal under multiple conditions or the amplitude difference between two conditions. In this experiment, two conditions are involved: condition one is that the signal appears, condition two is that the subject reacts. The contrast vector is designed as^1^; (4) short-distance channel measurements are added to GLM as covariates. In GLM, changes in Oxy-Hb concentration levels for each channel are simulated by convolution of a typical HRF with a binary time series containing stimulus timing.

To compare changes in Oxy-Hb concentrations across various environments, we organized data from the “Oxydata” folder, which contains part of the pre-processed information. Data from all participants within the same condition were pooled. The average Oxy-Hb concentration was calculated for each channel, yielding mean values for 19 channels (6 short-distance channels were excluded). These averaged concentrations were then subjected to Tukey’s HSD test at a 0.05 significance level to evaluate differences under different conditions. Additionally, to investigate the effects of temperature and humidity on the Oxy-Hb concentration among miners, multiple linear regression models were employed. Furthermore, an ANCOVA was performed to determine the independent effects of age on Oxy-Hb concentrations, apart from temperature and humidity.

To observe how the two ROIs differ in response to stimuli under different environmental conditions, we calculated the “Oxy\con1” data after GLM calculations. Beta regression coefficients were obtained as indicators of brain activity for each channel, averaged these data according to channel classification, and visualized the results. A Wilcoxon signed-rank test was used to examine the differences between the two ROIs in different conditions.

For the purpose of visualizing the Oxy-Hb time course, the time series of changes in Oxy-Hb levels were averaged across participants in each channel and in each condition. Because the stimulus duration varied in the millisecond range, we considered the stimulus presentation and its subsequent response to last 1 s, and a window of 5 s before and 15 s after the stimulus presentation was plotted on the graph to facilitate visual inspection.

## Results

We established three ANCOVA models to analyze the relationship between reaction time, error rate, Oxy-Hb concentrations and age respectively, so that the influence of age on these three variables can be effectively controlled. The ANCOVA analysis results are shown in Table 3.

**Table 3:** ANCOVA analysis results of the effects of age on RT, ER and Oxy-Hb.

	Reaction time				Error rate				Oxy-Hb concentrations (e-08)			
	Estimate	SE	T	*p*	Estimate	SE	T	*p*	Estimate	SE	T	*p*
(Intercept)	382.52	5.390	71.02	0	11.571	5.664	2.04	0.0527	4.1988	8.1704	− 0.514	0.6122
Age	− 0.2037	0.164	− 1.241	0.227	− 0.0788	0.172	− 0.456	0.652	− 3.2588	0.2491	1.3083	0.2037
R-squared	0.0628				0.00898				0.0693			
Adjusted R-squared	0.022				− 0.0341				0.0288			

SE stands for standard error.

The results above indicate that age has no significant impact on RT or ER or Oxy-Hb concentrations in the current data. Therefore, in subsequent analyses and reporting for this study, the influence of other factors on RT and ER and Oxy-Hb concentrations can be considered, instead of the influence of age.

### Behavioral results

The RT and ER under the four conditions were subjected to Tukey’s HSD test, the results are displayed in Table 4.

**Table 4:** Tukey’s HSD test results for reaction time and error rate.

Cond	Reaction time				Error rate			
	MD	LowerCI	UpperCI	p	MD	LowerCI	UpperCI	p
I vs. II	− 30.78	− 26.35	− 21.92	1.34e-27	− 14.66	− 12.19	− 9.73	3.77e-22
I vs. III	− 12.59	− 8.16	− 3.72	3.16e-05	− 2.57	− 0.10	2.36	0.9999
I vs. IV	− 47.57	− 43.14	− 38.72	0	− 19.56	− 17.09	− 14.63	2.52e-33
II vs. III	13.76	18.19	22.62	1.98e-17	9.63	12.09	14.56	4.78e-22
II vs. IV	− 21.22	− 16.79	− 12.36	1.24e-15	− 7.37	− 4.89	− 2.43	6.71e-06
III vs. IV	− 39.41	− 34.98	− 30.55	0	− 19.46	− 16.99	− 14.53	3.35e-33

Cond stands for conditions. MD stands for mean difference.

Based on Condition I, the pairwise comparisons among the environments concerning RT indices have attained a significant level in intergroup distinctions. This denotes that variations in either temperature or humidity exert a substantial impact on the participants’ RT. With regard to ER, significant differences were observed between environments with varying temperatures (Condition I vs. II, and Condition III vs. IV), indicating that temperature has a significant effect on ER. However, in environments with differing humidity (Condition I vs. III, and Condition II vs. IV), only the comparison between Condition II and IV achieved significance, while the difference between Condition I and III was not significant.

The analysis of RT and ER both indicated that temperature fluctuations have a more substantial impact on participants’ overall cognitive performance than humidity. An increase in temperature leads to significant disparities in both RT and ER, regardless of the humidity conditions. Conversely, an increase in humidity only affects cognitive performance at high temperatures.

Therefore, temperature may be the more dominant environmental factor in this experiment, capable of negatively impacting participants’ information processing speed and accuracy. To further validate the hypothesis that temperature has a more substantial effect on miners’ RT and ER than humidity, a multiple linear regression model was utilized to analyze RT and ER from the four groups.

By using temperature and humidity as independent variables, RT and ER serve as dependent variables in two separate multiple linear regression models. The models are constructed using MATLAB, with the results of both regressions shown in Table 5.

**Table 5:** Multiple liner regression results for reaction time and error rate.

	Reaction time				Error rate			
	Estimate	SE	T	*p*	Estimate	SE	T	*p*
Temperature	3.06	0.128	23.85	0.0281	1.46	0.072	20.41	0.0356
Humidity	0.42	0.043	9.75	0.0331	0.08	0.024	3.45	0.1292
R-squared	0.875				0.819			
Adjusted R-squared	0.873				0.814			

Regarding RT, the coefficient for temperature is 3.06, signifying that for every unit increase in temperature, the average RT slows down by 3.06 units. The standard error of temperature is 0.128, with t-value of 23.85 and *p* value less than 0.05. This indicates that the effect of temperature on RT is statistically significant. The coefficient for humidity is 0.42, which means that for each unit increase in humidity, the average RT slows down by 0.42 units. The standard error for humidity is 0.043, with t-value of 9.75 and *p* value less than 0.05, which also indicates that humidity has a statistically significant negative impact on RT. Concerning the ER: the coefficient estimate for temperature is 1.46, meaning that for each unit increase in temperature, the average ER rises by 1.46 units. The standard error for the temperature is 0.072, with t-value of 20.41 and *p* value less than 0.05. This suggests that the impact of temperature on ER is significant and positive. The coefficient estimate for humidity is 0.08, indicating that each unit increase in humidity results in an increase of 0.08 units in ER. The standard error for humidity is 0.024, with a t-value of 0.1292. Given that the *p* value is greater than 0.05, this implies that the effect of humidity on ER is not statistically significant, suggesting that in this experiment, variations in humidity do not have a statistically effect on ER.

### fNIRS results

#### Oxy-Hb Concentration

A Tukey’s HSD analysis was conducted on the Oxy-Hb concentration across all 19 channels in four conditions (Table 6).

**Table 6:** Tukey’s HSD test results for Oxy-Hb concentration.

Cond	Oxy-Hb concentration			
	MD (e-08)	Lower CI (e-08)	Upper CI (e-08)	P
I vs. II	− 5.4221	− 2.7058	0.010544	5.1272e-02
I vs III	− 1.7029	1.0134	3.7297	0.76055
I vs. IV	− 9.7069	− 6.9906	− 4.2743	2.1293e-08
II vs. III	1.0029	3.7192	6.4355	3.1813e-03
II vs. IV	− 7.0012	− 4.2849	− 1.5686	5.1463e-04
III vs. IV	− 17.20	− 8.0040	− 5.2877	4.0346e-09

Under varying conditions of temperature and humidity, significant changes in Oxy-Hb concentration were observed. Specifically, moving from 25 °C with 55% relative humidity (Condition I) or 35 °C with 55% relative humidity (Condition II) to 35 °C with 85% relative humidity (Condition IV) showed significant increase in oxyhemoglobin concentration (*p* < 0.00021293 and *p* < 0.000051, respectively). This emphasizes the pronounced impact of temperature increase in conjunction with high humidity on blood oxygenation.

To test the hypothesis that temperature impacts Oxy-Hb concentrations more significantly than humidity, a multiple linear regression model was developed. This model examines the effects of temperature and humidity on the oxyhemoglobin concentration across various channels. Using temperature and humidity as independent variables and oxyhemoglobin concentration as the dependent variable, the model was constructed in MATLAB. The regression results are displayed in Table 7.

**Table 7:** Multiple liner regression results for Oxy-Hb.

	Estimate	SE	T	*p*
Temperature	5.3549e-09	7.8877e-10	6.7889	2.5591e-09
Humidity	5.4524e-10	2.6292e-10	2.0737	0.0416
R-squared	0.408			
Adjusted R-Squared	0.392			

The regression analysis indicated that temperature significantly influences Oxy-Hb concentrations more than humidity. Temperature showed a substantial positive correlation with Oxy-Hb levels (*p* = 2.5591e-09), whereas humidity’s effect was smaller yet significant (*p* = 0.0416). Approximately 40% of Oxy-Hb variability is explained by these environmental factors, with temperature being the more dominant influence. This underscores the importance of temperature in physiological studies involving Oxy-Hb levels.

#### ROIs activation

MATLAB software was used to perform the Wilcoxon sign-rank test on the beta values within ROIs in the four cases (Table 8).

**Table 8:** Wilcoxon signed-rank tests results for two ROIs.

ROI	Cond	Mean (e-06)	Std (e-06)	Z			*p* value			Corrected *p* value		
				II	III	IV	II	III	IV	II	III	IV
A	I	6.84	3.61	− 1.83	–1.83	− 1.83	0.002	0.002	0.002	0.0117	0.0117	0.0117
	II	9.9	4.24		18.26	− 1.83		0.002	0.002		0.0117	0.0117
	III	8.08	4.49			− 1.83			0.002			0.0117
	IV	11.8	4.24									
B	I	5.58	3.67	− 1.83	− 1.83	− 1.83	0.002	0.002	0.002	0.0117	0.0117	0.0117
	II	8.28	4.18		18.26	− 1.83		0.002	0.002		0.0117	0.0117
	III	6.90	3.87			− 1.83			0.002			0.0117
	IV	10.0	4.87			–						

In order to avoid the “multiple test problem” that occurs when multiple comparisons are made, that is, the probability of false positives (false positives) increases, we used the Bonferroni correction method to adjust the Wilcoxon test results for multiple comparisons.

Comparing the initial and corrected P-values, it can be found that the correction process significantly raises the significance threshold and reduces the possibility of false positive results.

**Figure 5:**
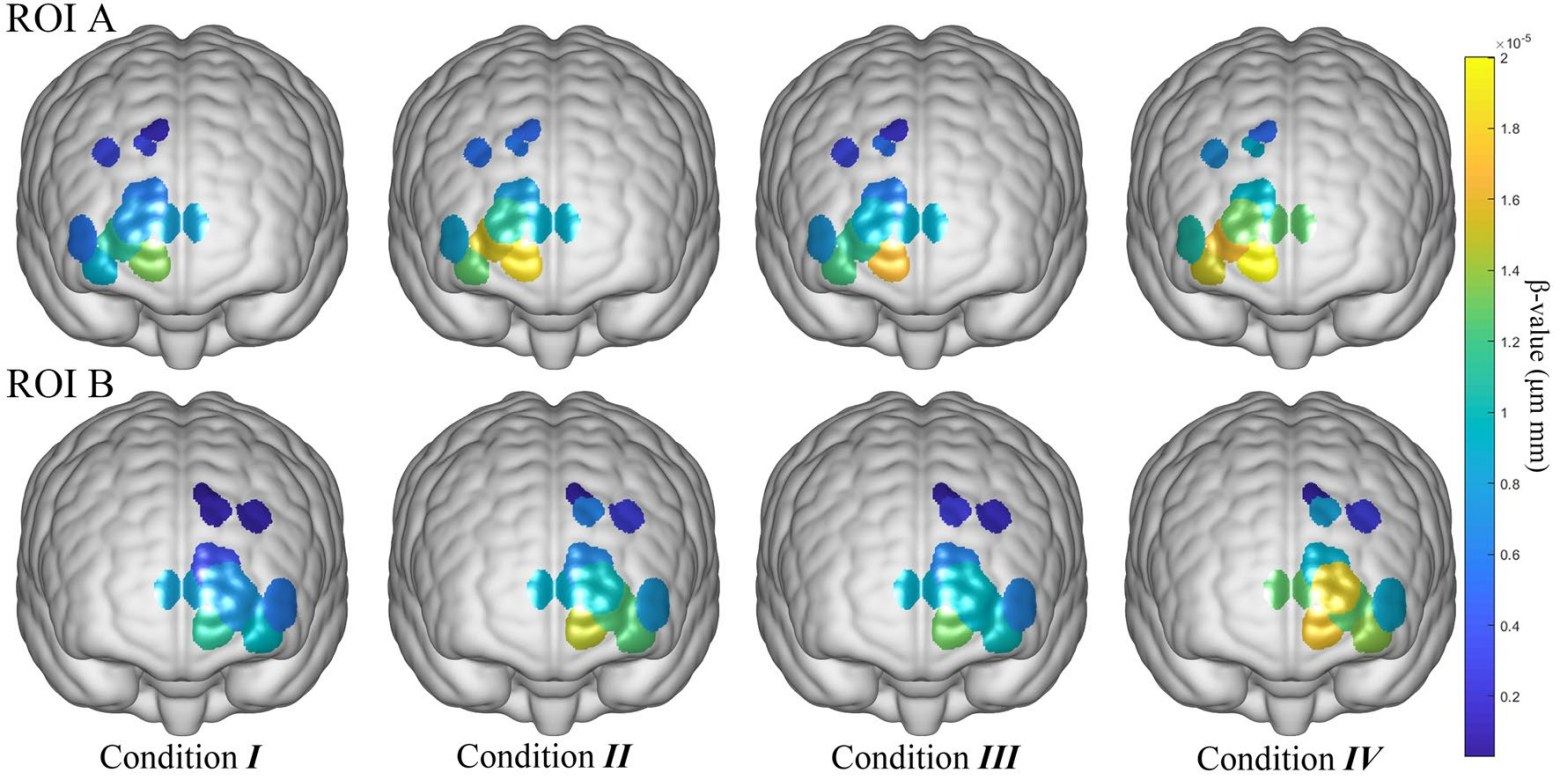
Visualization of the activation of ROIs under four conditions.

To visualize the level of ROI activation, use NIRS_KIT’s results visualization feature for 3D visualization, the results shown in Fig. 5.

The yellow color in the graph represents a higher level of activation, while the blue color indicates a lower level of activation. Based on the 3D plot, the effect of humidity on activation seems less significant than that of temperature, as evidenced by the corrected P-values in Table 8. Specifically, in ROI A, the *p* value of all comparisons show values less than 0.05, indicating significant changes. Although the corrected *p* values between condition I and II and IV are both 0.0117, the 3D plot shows that the activation in condition IV is notably higher than in condition II.

The Bonferroni correction results indicate that in ROI B, activation shows significant changes under all four conditions. However, in the 3D plot, it is evident that temperature has a greater impact on activation in ROI B, with activation in condition II being more prominent than in condition III. Additionally, the overall activation level in ROI B is lower than that in ROI A.

In summary, while the activation levels in ROI A and B increase with rising temperatures, an increase in humidity significantly affects only ROI B.

#### Data time course visualization

**Figure 6:**
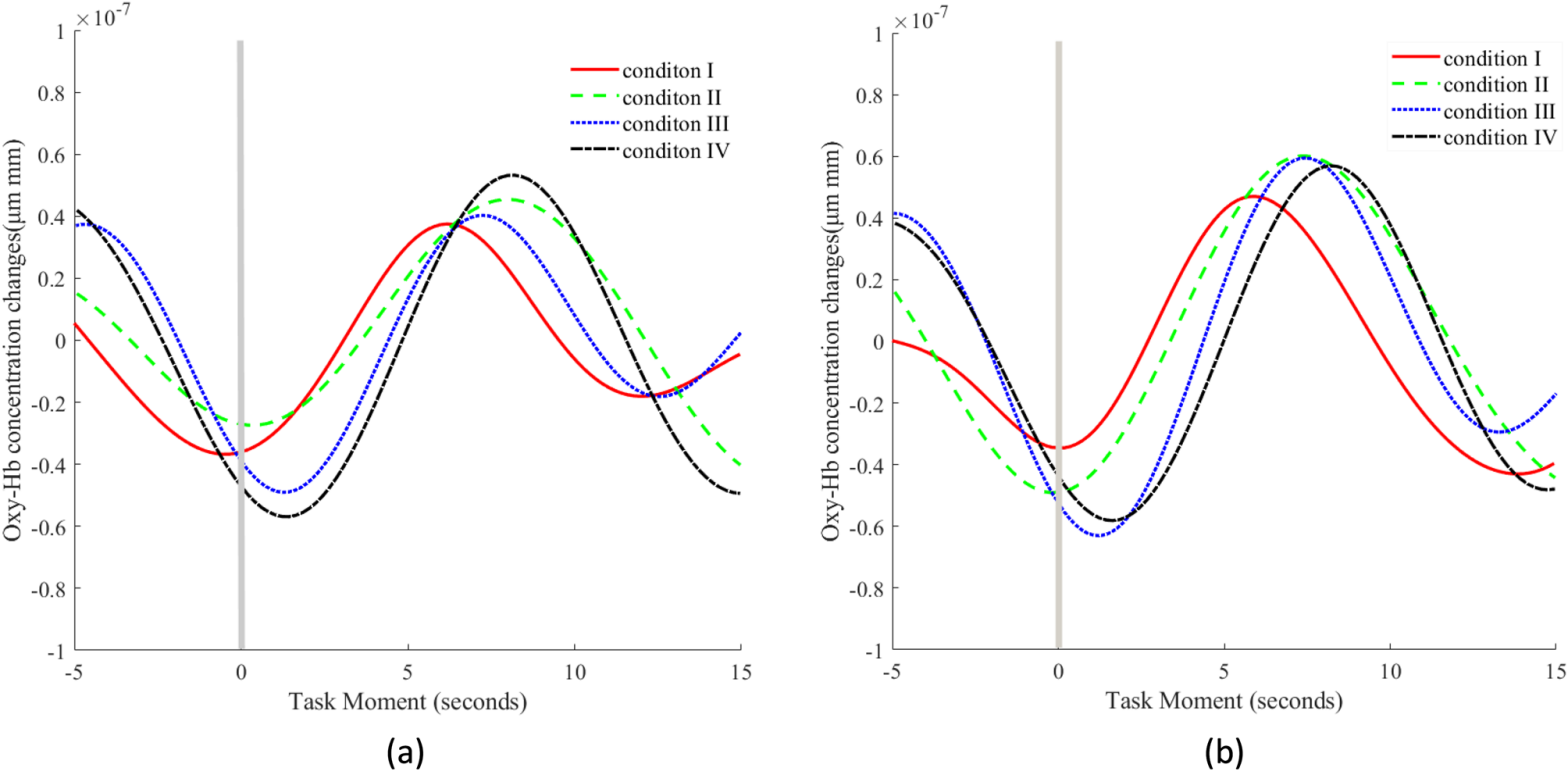
Oxy-Hb time courses. *Note* Averaged time course across all participants of Oxy-Hb concentration changes between 4 conditions in two ROIs. Gray vertical line represents stimulus presentation. (**a**) in ROI A (**b**) in ROI B.

The visualization of hemodynamic responses in PFC under varying environmental conditions revealed distinct differences in response time, peak time, peak amplitude, and recovery time between the right (ROI A) and left (ROI B) regions (Fig. 6). ROI B demonstrated consistently slower response times across all conditions, while ROI A exhibited quicker responses, especially under condition IV. Condition IV triggered the highest peak amplitude in ROI A, accompanied by the most delayed peak time compared to other conditions. In contrast, ROI B reached its peak more rapidly, though the peak amplitudes were less pronounced, particularly under condition IV. Following the peak, all conditions showed prolonged recovery times back to baseline levels. The most extended recovery was observed in ROI A under condition II. These results show a clear pattern of differential responsiveness in the bilateral PFC, with the right side showing heightened sensitivity and slower recovery under adverse conditions, which contrasts with the quicker and more stable responses observed in the left PFC.

## Discussion

This study employs fNIRS to investigate differences in miners’ alertness behavior data (response time, error rate) and fNIRS data (Oxy-Hb concentration, activation of ROIs and Oxy-Hb time courses) under varying conditions of temperature and humidity. To our knowledge, this study is the first to examine the impact of temperature and humidity conditions on miners’ neurocognitive functions from a combined perspective of neuroscience and behavioral science, with a particular focus on alertness.

Initially, we conducted an analysis of behavioral data, specifically focusing on the response time and error rates in the PVT experiment. Our study results indicate that reaction times and error rates exhibit significant variations under different temperature and humidity conditions, especially in condition IV, where the combined effect of temperature and humidity is very significant. In high-temperature and high-humidity environments, the error rate is highest and reaction time is longest, indicating that elevated temperatures and humidity can lead to impaired judgment in tasks requiring focused attention among miners. This finding aligns with the research reported by Zhao et al.^42^. This conclusion validates the synergistic effects of temperature and relative humidity, consistent with the findings presented by Shi et al.^43^. We also found that temperature has a more pronounced impact on reaction times and error rates than humidity.

In the second part, we focused on the ongoing brain activity, we conducted an analysis of fNIRS data, specifically focusing on the Oxy-Hb concentration, the activation of ROIs and Oxy-Hb time courses. Upon pairwise comparison of Oxy-Hb concentrations under various conditions, we discovered that Oxy-Hb under high temperature and high humidity conditions showed significant differences when compared to the other three conditions, whereas the differences among those three conditions were not statistically significant. This suggests that high temperature and humidity may lead to a decline in cognitive abilities, which is consistent with the findings of Zhang et al. ^44^. It also further corroborates the combined effect of temperature and humidity ^43^.

We analyzed the degree of influence of humidity and temperature on Oxy-Hb concentrations. The results revealed that, although the *p* values for both temperature and humidity were less than 0.05, indicating significant effects on Oxy-Hb, in comparison, each unit increase in temperature results in a greater change in Oxy-Hb concentration compared to each unit increase in humidity, signifying that temperature exerts a greater impact on Oxy-Hb concentrations.

To find the activation of ROIs, visualizations were utilized to depict the level of activation across the channels corresponding to two ROIs. It was observed that across all ROIs, the highest level of channel activation was consistently found under Condition IV. In two ROIs, both temperature and humidity exhibited significant effects on channel activation, with temperature having a more pronounced effect.

ROI A and B are part of the PFC, constituting a portion of the frontal attentional region, responsible for the allocation and maintenance of attentional resources^45^. The PFC is also considered a pivotal region of the brain, providing biased signals to other brain areas in order to activate neural pathways and establish the input and output patterns necessary for accomplishing specific tasks^46^. Hence, the changes in PFC activation are associated with an individual’s cognitive processes, information processing, and decision-making while performing tasks. Previous research has demonstrated a positive correlation between task difficulty and the activation level of the PFC^47^. In the context of this experiment, the increase in temperature and humidity leads to an elevated cognitive load. This is because an individual’s ability to sustain attention and process information from the environment is stronger in a relatively comfortable setting (Condition I), where the degree of brain activation is lower. The increase in cognitive load can be understood as an elevation in task difficulty. The experimental paradigm used in this study was the same, and participants performed the same tasks in different experimental environments. When the temperature and humidity were higher, participants needed to exert greater effort to complete the same tasks, which could be regarded as an increase in “workload” or an increase in the intensity of work, which poses additional attentional demands on the limited global workspace, thereby reducing the available resources for cognitive tasks. According to the global workspace theory, when both overall attentional resources are insufficient, the performance of cognitive tasks can deteriorate^48^. Under such circumstances, decreased focus or physical fatigue increases the likelihood of task failures or omissions, which, in practical production and daily life, can potentially lead to accidents.

Finally, we conducted a visual analysis of the time course of Oxy-Hb concentration changes. The observed differences in hemodynamic responses between the right and left prefrontal cortex under various environmental conditions reflect their specialized functional roles. The right PFC, responsible for processing negative stimuli and complex cognitive tasks, exhibited increased sensitivity under stressful conditions, particularly with high heat and humidity. This suggests an enhanced cognitive load and possibly emotional distress, emphasizing its role in managing challenging environments. Conversely, the left PFC demonstrated more consistent response times across different conditions, indicating a resilience to environmental changes. This consistency supports its role in regulating positive emotions and managing cognitive control tasks such as memory and semantic processing, suggesting a robust mechanism for maintaining cognitive performance and emotional stability. The temporal characteristics and their manifestation in specific brain regions noted in our study show similarities to those reported by Dosenbach et al.^35^, whose research highlighted the complex interactions within neural networks during executive control and emotional regulation. Our results further elaborate on this by showing that the right and left prefrontal cortex respond differently to environmental stress, illustrating a nuanced adaptation of these neural networks. This adaptive response underlines the specialized roles of different PFC regions in managing cognitive load and emotional responses appropriately according to the environmental demands.

## Conclusion

This study investigates the impact of varying temperatures and humidity on the alertness levels of miners during PVT task, employing fNIRS technology. The experiment controlled for additional variables that may affect the hemodynamic responses of the subjects, including illumination, wind speed, and noise levels.

The study found that there was a significant increase in cognitive load among the subjects because of the rising temperature and humidity, which was evident in their extended reaction times, higher error rates, and higher Oxy-Hb concentrations. Additionally, the effect of temperature on these parameters was greater than that of humidity. Within the three ROIs, the level of activation in the channels increased with the rise in temperature and humidity. Notably, the response in the right PFC region was particularly pronounced, and this is also reflected in the time course of Oxy-Hb.

The findings of this study have provided new insights into the impact of underground coal mine environments on workers’ cognitive abilities in China. In future research, the application of fNIRS technology can be employed to investigate variations in the functional connectivity and local efficiency of miners’ brain under different environmental conditions. This can provide technical and theoretical support for determining the optimal underground working environment.

## Data Availability

The data that support the findings of this study are available from the corresponding author upon reasonable request.

## References

[1] Wang J, Xie H, Liu J, Wu L, Ren S, Jiang P, Zhou H (2018). Theoretical and technical conception of coal development and utilization with near-zero ecological and environmental impact. J. China Coal Soc..

[2] Guo Q, Cai M, Wu X, Xi X, Ma M, Zhang J (2022). Development strategy for intelligent multi-field deep mining of metal mines towards 2035. Chin. J. Eng..

[3] Wang L, Liang Y, Luo H (2018). Progress and prospects of theoretical research on thermodynamic disasters in Chinese mines. Coal Sci. Technol..

[4] Song X, Xie Z (2013). Research on mine cooling measures for Zhangshuanglou coal mine. Int. Symp. Mine Saf. Sci. Eng..

[5] Zhongpeng X (2012). Distribution law of high temperature mine’s thermal environment parameters and study of heat damage’s causes. Procedia Eng..

[6] Li Z (2021). Impact of the water evaporation on the heat and moisture transfer in a high-temperature underground roadway. Case Stud. Therm. Eng..

[7] Xie H-P (2017). Research framework and anticipated results of deep rock mechanics and mining theory. Adv. Eng. Sci..

[8] He M, Xie H, Peng S, Jiang Y-D (2005). Study on rock mechanics in deep mining engineering. Chin. J. Rock Mech. Eng..

[9] Manchao He, Pingye G (2013). Thermodynamic effects of deep rock masses and temperature control countermeasures. Chin. J. Rock Mech. Eng..

[10] Legault G (2011). Sleep and heat related changes in the cognitive performance of underground miners: a possible health and safety concern. Minerals.

[11] Costa C (2020). Night shift work in resident physicians: Does it affect mood states and cognitive levels?. J. Affect. Disord..

[12] Pereira H (2021). The impact of shift work on occupational health indicators among professionally active adults: A comparative study. Int. J. Environ. Res. Public Health.

[13] Bell C, Provins K, Hiorns R (1964). Visual and auditory vigilance during exposure to hot and humid conditions. Ergonomics.

[14] Norin F, Wyon DP (1992). Driver Vigilance-the Effects of Compartment Temperature.

[15] Song X (2017). Resting-state BOLD oscillation frequency predicts vigilance task performance at both normal and high environmental temperatures. Brain Struct. Funct..

[16] Te Lindert BH, Van Someren EJ (2018). Skin temperature, sleep, and vigilance. Handb. Clin. Neurol..

[17] Fronczek R (2008). Manipulation of core body and skin temperature improves vigilance and maintenance of wakefulness in narcolepsy. Sleep.

[18] Raymann RJ, Van Someren EJ (2007). Time-on-task impairment of psychomotor vigilance is affected by mild skin warming and changes with aging and insomnia. Sleep.

[19] Lara T, Molina E, Madrid JA, Correa Á (2018). Electroencephalographic and skin temperature indices of vigilance and inhibitory control. Psicol. J.

[20] Herold F, Wiegel P, Scholkmann F, Müller NG (2018). Applications of functional near-infrared spectroscopy (fNIRS) neuroimaging in exercise–cognition science: A systematic, methodology-focused review. J. Clin. Med..

[21] Berkman ET, Falk EB (2013). Beyond brain mapping: Using neural measures to predict real-world outcomes. Curr. Dir. Psychol. Sci..

[22] Ferrari M, Quaresima V (2012). A brief review on the history of human functional near-infrared spectroscopy (fNIRS) development and fields of application. Neuroimage.

[23] Scholkmann F (2014). A review on continuous wave functional near-infrared spectroscopy and imaging instrumentation and methodology. Neuroimage.

[24] Tak S, Ye JC (2014). Statistical analysis of fNIRS data: A comprehensive review. Neuroimage.

[25] Nakano T, Watanabe H, Homae F, Taga G (2009). Prefrontal cortical involvement in young infants’ analysis of novelty. Cereb. Cortex.

[26] Taga G, Asakawa K, Maki A, Konishi Y, Koizumi H (2003). Brain imaging in awake infants by near-infrared optical topography. Proc. Natl. Acad. Sci..

[27] Liu T, Pelowski M, Pang C, Zhou Y, Cai J (2016). Near-infrared spectroscopy as a tool for driving research. Ergonomics.

[28] Hu M, Shealy T, Hallowell M, Hardison D (2018). Advancing construction hazard recognition through neuroscience: Measuring cognitive response to hazards using functional near infrared spectroscopy. Constr. Res. Congress.

[29] Dehais, F., et al., Monitoring pilot’s cognitive fatigue with engagement features in simulated and actual flight conditions using an hybrid fNIRS-EEG passive BCI, in *2018 IEEE International Conference on Systems, Man, and Cybernetics (SMC)* (IEEE, 2018), pp. 544–549

[30] Wang P (2018). Impact of humidity in underground workplaces of coal mines on workers’ physical and mental health. Occup. Health.

[31] Hall RW (2008). Long-term deficits of preterm birth: Evidence for arousal and attentional disturbances. Clin. Neurophysiol..

[32] Hiroyasu, T., Fukushima, A., Yokouchi, H., Differences in blood flow between auditory and visual stimuli in the psychomotor vigilance task and GO/NOGO task, in *2012 Annual International Conference of the IEEE Engineering in Medicine and Biology Society* (IEEE, 2012), pp. 1466–1469 10.1109/EMBC.2012.6346217 23366178

[33] Borragán G, Guerrero-Mosquera C, Guillaume C, Slama H, Peigneux P (2019). Decreased prefrontal connectivity parallels cognitive fatigue-related performance decline after sleep deprivation. An optical imaging study. Biol. Psychol..

[34] Drummond SP (2005). The neural basis of the psychomotor vigilance task. Sleep.

[35] Dosenbach NU (2007). Distinct brain networks for adaptive and stable task control in humans. Proc. Natl. Acad. Sci..

[36] Oostenveld R, Praamstra P (2001). The five percent electrode system for high-resolution EEG and ERP measurements. Clin. Neurophysiol..

[37] Hou X (2021). NIRS-KIT: A MATLAB toolbox for both resting-state and task fNIRS data analysis. Neurophotonics.

[38] Cope M, Delpy DT (1988). System for long-term measurement of cerebral blood and tissue oxygenation on newborn infants by near infra-red transillumination. Med. Biol. Eng. Comput..

[39] Fishburn FA, Ludlum RS, Vaidya CJ, Medvedev AV (2019). Temporal derivative distribution repair (TDDR): A motion correction method for fNIRS. Neuroimage.

[40] Yücel MA (2021). Best practices for fNIRS publications. Neurophotonics.

[41] Strangman G, Franceschini MA, Boas DA (2003). Factors affecting the accuracy of near-infrared spectroscopy concentration calculations for focal changes in oxygenation parameters. Neuroimage.

[42] Zhao J, Zhu N, Lu S (2009). Productivity model in hot and humid environment based on heat tolerance time analysis. Build. Environ..

[43] Shi X, Zhu N, Zheng G (2013). The combined effect of temperature, relative humidity and work intensity on human strain in hot and humid environments. Build. Environ..

[44] Zhang F (2017). The effects of higher temperature setpoints during summer on office workers’ cognitive load and thermal comfort. Build. Environ..

[45] Nogueira MG (2022). Differences in brain activity between fast and slow responses on psychomotor vigilance task: An fNIRS study. Brain Imaging Behav..

[46] Miller EK, Cohen JD (2001). An integrative theory of prefrontal cortex function. Annu. Rev. Neurosci..

[47] Fairclough SH, Burns C, Kreplin U (2018). FNIRS activity in the prefrontal cortex and motivational intensity: Impact of working memory load, financial reward, and correlation-based signal improvement. Neurophotonics.

[48] Hocking C, Silberstein RB, Lau WM, Stough C, Roberts W (2001). Evaluation of cognitive performance in the heat by functional brain imaging and psychometric testing. Comp. Biochem. Physiol. Part A Mol. Integr. Physiol..

